# Methimazole-induced myositis: a case report and review of the literature

**DOI:** 10.1530/EDM-13-0008

**Published:** 2013-08-30

**Authors:** R Bou Khalil, M Abou Salbi, S Sissi, N El Kara, E Azar, M Khoury, G Abdallah, J Hreiki, S Farhat

**Affiliations:** 1 Department of EndocrinologySaint Georges University Medical Center, University of BalamandBeirutLebanon; 2 Department of Family MedicineSaint Georges University Medical Center, University of BalamandBeirutLebanon; 3 Department of Infectious DiseasesSaint Georges University Medical Center, University of BalamandBeirutLebanon; 4 Department of General SurgerySaint Georges University Medical Center, University of BalamandBeirutLebanon; 5 Department of PathologySaint Georges University Medical Center, University of BalamandBeirutLebanon; 6 Department of GastroenterologySaint Georges University Medical Center, University of BalamandBeirutLebanon

## Abstract

Methimazole is an anti-thyroid drug commonly used to treat hyperthyroidism and is a relatively safe medication. Several side effects have been reported and usually develop within 3 months of therapy. Well-known adverse reactions include agranulocytosis, hepatitis, skin eruptions, and musculoskeletal complaints such as myalgia, arthralgia, and arthritis. So far, myositis secondary to carbimazole was described in the context of a lupus-like syndrome or other rare cases of anti-neutrophil cytoplasmic antibodies-associated vasculitis. Methimazole-induced myositis occurring independently of such reactions was rarely stated. We report a patient with hyperthyroidism who, early after therapy with methimazole, developed hepatitis, eosinophilia, and fever that resolved completely after stopping the medication as well as a delayed onset of biopsy-proven eosinophilic myositis and fasciitis of gluteal muscles that resolved eventually without any additional therapy. Therefore, we raise the awareness regarding a rare side effect of methimazole: myositis.

**Learning points:**

- Several differential diagnoses arise when managing a hyperthyroid patient with muscle complaints.
- Both hyperthyroidism and methimazole are associated with myositis.
- Methimazole-induced myositis is a rare clinical entity.
- Resolution of symptoms may occur after stopping methimazole.

## Background

One of the frequently used medications for hyperthyroidism is methimazole. Musculoskeletal complaints are common in thyroid disorders and are also described as side effects to anti-thyroid drugs. Myositis is not a classical feature in either situation. We present a case of myositis in a hyperthyroid patient that started shortly after initiating methimazole and resolved after its withdrawal, with a biopsy-proven eosinophilic myositis and lack of vasculitis. Thus, we describe an unusual case of myositis due to methimazole that was reversed after drug withdrawal.

## Case presentation

A 29-year-old male patient who has been previously healthy, on no medications, presented for weight loss during the last 6 months. Graves' disease was diagnosed (Table 1). He was started on propranolol 60 mg/day and methimazole 20 mg/day. Two weeks later, he developed a febrile illness. These results revealed a mixed pattern of disturbed liver function tests (cytolytic and cholestatic) and hypereosinophilia (Table 1). Methimazole was stopped. Complementary work-up for viral hepatitis including hepatitis A, B, and C; Epstein Barr virus; and cytomegalovirus, as well as serologies for salmonella and brucella, were negative. The fever subsided a few days later without additional therapy. Two weeks after stopping methimazole, the fever reappeared associated with left gluteal swelling and mild erythema over the involved area. He was hospitalized. Laboratory tests revealed hypereosinophilia, increased creatine phosphokinase, and persistent elevation of liver enzymes (Table 1). Blood and urine cultures were negative. Perinuclear for anti-neutrophil cytoplasmic antibodies (ANCA), cytoplasmic ANCA, ENA profile (anti-Sm, anti-RNP, anti-SS-A, anti-SS-B, anti-Jo-1, anti-SCL-70, anti-CENP B) and serology for trichinella were also negative except for positive double-stranded DNA (19, normal<10).

**Table 1:** Laboratory values

**Laboratory tests**	**Reference range**	**Patient's values**			
At diagnosis of hyperthyroidism	Two weeks after starting methimazole (onset of fever)	Two weeks after stopping methimazole (on admission to the hospital)	Two months after stopping methimazole
TSH	0.35–4.94	<0.005		0.0001	<0.0005
Free T_4_	0.7–1.48 ng/dl	2.6 ng/dl		1.81 ng/dl	24 pmol/l
12–22 pmol/l				
Free T_3_	1.71–3.71 pg/ml	16.7 pg/ml		4.93 pg/ml	6.81 pmol/l
3.0–6.8 pmol/l				
TRAB	<2	9			
GGT	8–61		391	124	59
SGOT	0–40		52	97	36
SGPT	0–41		136	64	37
Alkaline phosphatase	40–129		440	132	148
Bilirubin	Total: 0–1.1		Total: 1.25	Total: 0.86	
Direct: 0–0.3		Direct: 0.65	Direct: 0.39	
Creatine phosphokinase	24–190			755	147
WBC	4000–10 000 cells/μl		6300	7500	6400
Absolute eosinophil count			630	2250	118
ESR	–			38	

TRAB, thyroid receptor antibodies; GGT, gamma-glutamyl transpeptidase; SGOT, aspartate transaminase; SGPT, alanine aminotransferase; WBC, white blood count; ESR, erythrocyte sedimentation rate.

**Figure 1:**
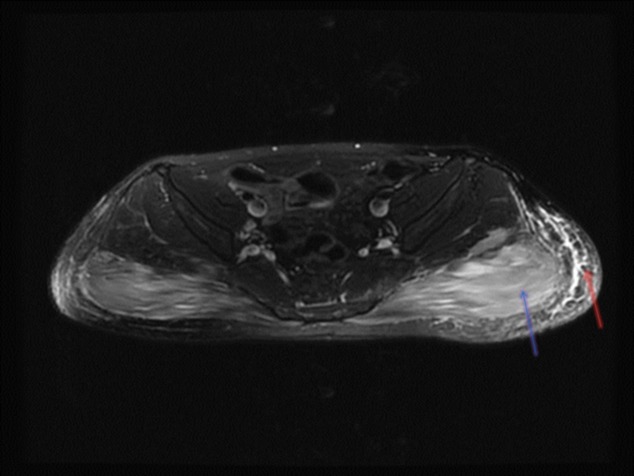
Magnetic resonance imaging of the pelvis. Signs of inflammation of the gluteus maximus muscles bilaterally, more so on the left (blue arrow), and the surrounding fascia (red arrow) are indicated.

Two weeks after stopping methimazole, magnetic resonance imaging of the pelvis performed upon hospitalization showed evidence of myositis in the gluteus maximus muscles bilaterally, much more marked on the left, associated with fasciitis (Fig. 1).

**Figure 2:**
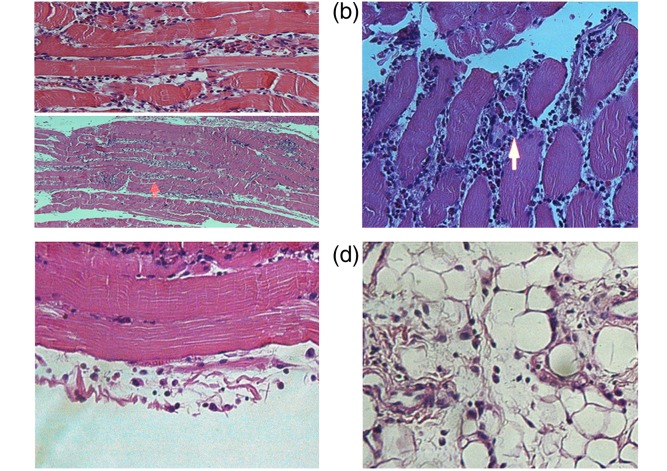
Pathology of the muscle biopsy, 2 weeks after stopping methimazole. (a) Dense eosinophilic infiltration of the myofibers associated with necrosis (red arrow). (b) Dense mixed inflammatory cell infiltrate with associated myofiber necrosis and regeneration (white arrow). (c) Minimal perimysial inflammatory cell infiltrate of plasma cells, eosinophils, and lymphocytes. (d) Fibrofatty tissue with minimal inflammatory cell infiltrate, identical to the previously described one.

A biopsy taken from the affected gluteus muscle showed dense diffuse perimysial and endomysial eosinophilic infiltrates (Fig. 2). No evidence of vasculitis or parasites was seen. The diagnosis of focal eosinophilic myositis was made.

## Treatment

The patient received only symptomatic treatment with nonsteroidal anti-inflammatory drugs. The fever subsided. Rapid clinical and laboratory improvements were evident. After 3 days of hospitalization, he was discharged solely on β-blockers.

## Outcome and follow-up

The laboratory results improved progressively after stopping the drug, with further decrease in the creatine kinase and the liver function tests. The symptoms resolved completely within 3 weeks. He returned 2 months later for radioactive iodine ablation. Four months after his last visit, the patient remains symptom free.

## Discussion

Muscle complaints are frequent in patients with thyroid disease. Hyperthyroidism is associated with proximal myopathy, hypothyroidism can cause creatine phosphokinase elevation, and rare cases of polymyositis have been described with thyroid dysfunction (1). Thyrotoxic myopathy is painless, the creatine phosphokinase is usually normal, and the muscle biopsy findings are not specific, including marked muscle fiber degeneration and atrophy (1). On the other hand, our patient had no evidence of hypothyroidism that might have accounted for the elevated creatine phosphokinase. As for polymyositis associated with Graves' disease, it is uncommon and occurs in the background of a positive family history of autoimmune diseases and positive antinuclear antibodies (2). The muscle biopsy characteristically shows marked atrophy with a predominantly lymphocytic infiltrate (1). Our patient, however, had evidence of eosinophilic infiltrates on the biopsy.

Parasitic infections are among the most common differential diagnoses of eosinophilic myositis (Table 2). Serology for trichinella, which is relatively more common than other parasites, was negative. In addition, other parasitic infections like hydatidosis, cysticercosis, toxocariasis, and toxoplasmosis (3) would probably have been seen on biopsy. There was no evidence of either neoplasm or trauma, as both can cause eosinophilic pseudo tumor (4).

**Table 2:** Differential diagnosis of eosinophilic myositis

****
Parasitic infection
Neoplasm
Trauma
Multisystem diseases
Hypereosinophilic syndrome
Idiopathic eosinophilic myositis

Eosinophilic myositis may be a feature of multisystem diseases like dermatomyositis, polyarteritis nodosa, and rheumatoid arthritis, but in this case, eosinophilia is rare (4) and the biopsy findings will show prominent vasculitis, which was not present in our case. Other rare features are hypereosinophilic syndrome characterized by hypereosinophilia with multisystem involvement and idiopathic eosinophilic myositis (Table 2). The former, however, is characterized by polymyositis rather than focal myositis (4).

In our case, the temporal relationship between the myositis and the introduction of methimazole cannot be ignored. Although the positivity of the double-stranded DNA can point toward an underlying lupus that has been unrecognized, the reversibility of symptoms after stopping methimazole and the marked eosinophilia are characterizing this episode as a drug reaction.

There are very few cases reporting the association between carbimazole therapy and myositis (2). The underlying mechanism is not well understood. Some related it to a direct effect of carbimazole on muscle (5) and others related it to a rapid decrease in thyroid hormones resulting in a relative local hypothyroid state within the muscle, which may contribute to the creatine phosphokinase elevation (5). The latter, however, does not explain the eosinophilic myositis nor the associated fasciitis. Murata *et al*. (6) showed that muscle damage in eosinophilic myositis is mediated by local accumulation of eosinophils and subsequent production of interleukin 5. A fourth suggested mechanism would be an immune-mediated reaction inducing a lupus-like syndrome (7).

Cases of myositis were described with thionamides in which there was an immune-mediated mechanism, with either drug-induced lupus (7) (8) or ANCA-positive vasculitis (9). Thereby, the onset of fever, rash, hepatitis, and myositis that were mild and subsided after methimazole withdrawal can be referred to a lupus-like syndrome. Other than anti-thyroid drugs, β-blockers have also been implicated in a lupus-like reaction (7), but our patient had complete resolution of symptoms while he was still on β-blockers. In drug-induced lupus, the symptoms usually appear within a month of starting therapy. Criteria for diagnosis are history of drug exposure, no history of systemic lupus erythematous prior to the episode, positive antinuclear antibodies (7) (8), and a rapid fall in serologies after stopping the drug (7). Antibodies to double-stranded DNA are detectable in <5% of drug-induced lupus (7). In our case, the patient could have drug-induced lupus as shown by the positive anti-double-stranded DNA but the marked eosinophilia, the presence of eosinophilic myositis, and the absence of vasculitis on biopsy pointed toward a different mechanism responsible for the myositis than a simple drug-induced lupus. Unfortunately, the antinuclear antibodies were not available initially but an evaluation done later showed a low titer of 1/80. Similarly, the ANCA were negative and the muscle biopsy did not show vasculitis.

In conclusion, methimazole is a relatively safe medication. Myositis is an infrequently encountered side effect of which physicians should be aware in a hyperthyroid patient with muscle complaints. Early diagnosis allows prompt withdrawal of the offending drug and resolution of symptoms.

## Patient consent

Written informed consent was obtained from the patient/patient's mother for publication of this case report.

## Author contribution statement

R Bou Khalil and M Abou Salbi were involved in data collection, patient care, and follow-up. S Sissi, N El Kara, and G Abdallah were involved in data collection and hospital care. E Azar, M Khoury, and S Farhat were involved in patient assessment, diagnosis, and treatment. J Hreiki was involved in the pathology evaluation and report.
